# Multiclass cancer classification in fresh frozen and formalin-fixed paraffin-embedded tissue by DigiWest multiplex protein analysis

**DOI:** 10.1038/s41374-020-0455-y

**Published:** 2020-06-29

**Authors:** Teresa Bockmayr, Gerrit Erdmann, Denise Treue, Philipp Jurmeister, Julia Schneider, Anja Arndt, Daniel Heim, Michael Bockmayr, Christoph Sachse, Frederick Klauschen

**Affiliations:** 1 Institute of Pathology, Charité – Universitätsmedizin Berlin, Corporate Member of Freie Universität Berlin, Humboldt-Universität zu Berlin and Berlin Institute of Health, Berlin, Germany; 2 NMI TT Pharmaservices, Berlin, Germany; 3 Central Biobank Charité (ZeBanC), Berlin, Germany; 4 German Cancer Consortium (DKTK), Partner Site Berlin, and German Cancer Research Center (DKFZ), Heidelberg, Germany; 5 Department of Pediatric Hematology and Oncology, University Medical Center Hamburg-Eppendorf, Hamburg, Germany; 6 Research Institute Children’s Cancer Center Hamburg, Hamburg, Germany

**Keywords:** Proteomic analysis, Cancer of unknown primary

## Abstract

Histomorphology and immunohistochemistry are the most common ways of cancer classification in routine cancer diagnostics, but often reach their limits in determining the organ origin in metastasis. These cancers of unknown primary, which are mostly adenocarcinomas or squamous cell carcinomas, therefore require more sophisticated methodologies of classification. Here, we report a multiplex protein profiling-based approach for the classification of fresh frozen and formalin-fixed paraffin-embedded (FFPE) cancer tissue samples using the digital western blot technique DigiWest. A DigiWest-compatible FFPE extraction protocol was developed, and a total of 634 antibodies were tested in an initial set of 16 FFPE samples covering tumors from different origins. Of the 303 detected antibodies, 102 yielded significant correlation of signals in 25 pairs of fresh frozen and FFPE primary tumor samples, including head and neck squamous cell carcinomas (HNSC), lung squamous cell carcinomas (LUSC), lung adenocarcinomas (LUAD), colorectal adenocarcinomas (COAD), and pancreatic adenocarcinomas (PAAD). For this signature of 102 analytes (covering 88 total proteins and 14 phosphoproteins), a support vector machine (SVM) algorithm was developed. This allowed for the classification of the tissue of origin for all five tumor types studied here with high overall accuracies in both fresh frozen (90.4%) and FFPE (77.6%) samples. In addition, the SVM classifier reached an overall accuracy of 88% in an independent validation cohort of 25 FFPE tumor samples. Our results indicate that DigiWest-based protein profiling represents a valuable method for cancer classification, yielding conclusive and decisive data not only from fresh frozen specimens but also FFPE samples, thus making this approach attractive for routine clinical applications.

## Abstract (web summary)

Proteomic profiling may contribute to the analysis and classification of cancer. The authors applied the digital western blot technique DigiWest with a panel of 102 proteins and phosphoproteins in combination with a machine learning algorithm to classify the tissue origin of five common cancer types in fresh frozen and formalin-fixed paraffin-embedded tissue. DigiWest profiling represents a valuable method for cancer classification, yielding conclusive and decisive data, thus making this approach attractive for routine clinical applications.

## Introduction

Precise cancer diagnostics is crucial for the selection of the appropriate treatment and estimation of prognosis. Tumor classification includes the tissue of origin (histological type and primary site), as well as the grade and stage of cancer. Besides clinical and radiographic data, the pathological examination is decisive in cancer diagnostics. Histomorphology complemented by immunohistochemistry allows for the determination of the tissue of origin in many cases. However, in certain instances, such as in squamous cell carcinomas, or when tumors have lost their specific features, this approach fails, and the tumor types cannot be determined by conventional methods. Moreover, in cancers of unknown primary (CUP), which account for 3–5% of all malignant epithelial tumors, no primary tumor can be identified even after extensive diagnostic work-up [1]. CUP are often adenocarcinomas, and more than half of the primaries found in autopsy arose in the lung or the pancreas [2, 3]. Furthermore, patients sometimes develop more than one cancer, which makes it difficult to differentiate metastasis from a second primary tumor. For example, primary squamous cell carcinomas of the lung and metastases from head and neck squamous cell carcinomas (HNSC) often share a similar morphology and can, therefore, hardly be distinguished from each other [4].

Through the advances in molecular techniques, sophisticated diagnostic approaches have been developed and applied to cancer classification. Several studies describe the classification of tumors according to their tissue of origin using gene expression [5–7], microRNA [8, 9], and, more recently, DNA methylation [10, 11] profiling. Tissue-based protein profiling constitutes a further promising approach for cancer classification, as tumor types are characterized by specific protein profiles [12]. Proteins are the principal effector molecules in a cell, and their function can be influenced by posttranslational modifications such as phosphorylation, acetylation, glycosylation, or sulphation [13]. Proteomic approaches have been successfully applied to differentiate cancer from nonmalignant tissue [14–16] or for the pairwise distinction of tumor types or subtypes [17–19]. However, so far, only a few studies have been conducted to classify multiple tumor types based on protein profiles in tissue samples [20–22].

Mass spectrometry is a powerful method for comprehensive proteomics, but is associated with high initial and operating costs, requires specially trained staff and has not yet been implemented routinely in diagnostics [23]. Besides mass spectrometry, diverse targeted antibody-based techniques have been established for protein analysis. Immunohistochemistry is widely used for standard diagnostic purposes and enables protein identification and quantification in histological sections [23]. Western blots facilitate immunodetection according to molecular weight [24]. However, both methods are inconvenient for the analysis of a high number of proteins and require a relatively large amount of tissue. In contrast, reverse phase protein arrays (RPPA) and the DigiWest method are more suitable for the parallel measurement of multiple proteins [25, 26]. DigiWest relies on classical western blotting and combines it with bead-based multiplexing, which allows for the simultaneous measurement of 80–800 proteins in samples even with low amounts of material [26]. Furthermore, DigiWest shows a similar sensitivity, reproducibility, and signal linearity as a high-end western blot system [26]. It has been effectively used in the analysis of signaling pathways and for the verification of biomarker candidates [27–31].

Fresh frozen samples are often preferred for molecular analysis, as macromolecules are preserved without cross-links [32], but the availability of fresh frozen samples is often limited as their collection is laborious, expensive, and necessitates special logistics. Hence, in routine diagnostic pathology, tissue samples are fixed in formalin and embedded in paraffin for preservation. Formaldehyde induces cross-links among proteins or between proteins and nucleic acids, thereby preserving the tissue morphology adequately [33]. This ensures good quality for histological examination, but cross-links may impair the immunoreactivity of proteins by modifying their conformation and altering or masking the epitope [34]. It is also challenging to extract full-length proteins from formaldehyde-fixed tissue [35]. Different protocols have already been developed to analyze formalin-fixed paraffin-embedded (FFPE) samples by immunohistochemistry, western blotting, mass spectrometry, and RPPA [36–39].

In this study, we established FFPE sample extraction protocols suitable for DigiWest protein profiling, tested over 600 antibodies for their suitability on FFPE tissue, and identified antibodies yielding comparable results in FFPE and fresh frozen tissue. We showed that DigiWest multiplex protein profiles can be used to predict the tissue of origin of five different cancer types, including HNSC, lung squamous cell carcinomas (LUSC), lung adenocarcinomas (LUAD), colorectal adenocarcinomas (COAD), and pancreatic adenocarcinomas (PAAD) in both fresh frozen and FFPE tissue.

## Materials and methods

### Sample acquisition and preparation

A set of 25 paired fresh frozen and FFPE tumor samples, as well as an independent validation cohort of 25 FFPE tumor samples, were acquired from the archive of the Institute of Pathology of the Charité University Hospital Berlin, Germany. Informed consent was obtained from all patients in accordance with standard institutional guidelines. The samples were all primary tumors.

The set of 25 paired fresh frozen and FFPE tumor samples contained five HNSC, five LUSC, five LUAD, five COAD, and five PAAD. Tumors with different histological grades were included (1 well, 18 moderately, 2 moderately to poorly, and 4 poorly differentiated) to represent tumors that would occur in a realistic clinical setting. Tumor cell content was assessed by a board-certified pathologist based on hematoxylin-eosin-stained slides and was determined to be at least 30% in fresh frozen and at least 40% in FFPE samples. The average tumor cell content was 70% in fresh frozen and 68% in FFPE samples.

The independent validation cohort consisted of 25 FFPE tumor samples, also with five cases per tumor type (HNSC, LUSC, LUAD, COAD, and PAAD). Of these, 18 tumor samples were moderately and seven poorly differentiated. The tumor cell content was at least 40% and averaged 68%.

Fresh frozen samples used for DigiWest analysis were collected after surgical resection, snap-frozen in liquid nitrogen, and stored at −80 °C. The cold ischemic time was measured in 21 of 25 samples with a median of 14 min. They were cut in slices of 15 µm thickness at −20 °C using a cryostat (Leica Biosystems, Wetzlar, Germany). Tissue slices were lysed in CLB1 lysis buffer (10 µl lysis buffer/mg tissue) containing PhosSTOP inhibitor cocktails (Roche Diagnostics GmbH) for 30 min in a thermomixer (4 °C, 1400 rpm). Samples were subsequently centrifuged for 5 min at 4 °C and 18,200 *g* (Eppendorf, Hamburg, Germany). The supernatant was collected, divided into three aliquots, and stored at −80 °C. The total protein concentration was measured using Coomassie Plus (Bradford) Assay Kit (Thermo Scientific, Rockford, USA). All samples had a protein concentration of more than 1 mg/ml.

FFPE samples were cut in 15 µm thick curls. When needed, tumor-rich areas were manually macro-dissected to ensure a tumor cell content of at least 40%. For the extraction of proteins from FFPE curls, the Qproteome FFPE Tissue kit (Qiagen, Hilden, Germany) with its Heptan-based protocol was used according to the manufacturer’s recommendations. The resulting protein lysates were further purified with the 2-D Clean-Up Kit (GE Healthcare, Chicago, USA) according to the vendor’s protocol. The resulting protein pellets were re-suspended in LDS buffer containing 212 mM Tris HCL, 282 mM Tris base, 4% LDS (w/v), 1.01 mM EDTA and supplemented with 50 mM DTT (Invitrogen, Carlsbad, USA). Protein concentrations were determined using the 660 nm assay with IDCR (Invitrogen, Carlsbad, USA).

Protein concentrations of both fresh frozen and FFPE lysates were then adjusted by SDS PAGE, employing Coomassie Fluor Orange Protein Gel Stain (Invitrogen, Carlsbad, USA) according to the vendor’s protocol, including an internal protein lysate standard to optimize sample loading for DigiWest.

### DigiWest multiplex protein analysis

DigiWest assays were performed as published (see [26] for details). In brief, for the initial analysis of 634 antibodies, 2 × 20 µg of total protein per sample was loaded on an SDS-polyacrylamide gel (20 µg/lane) and size-separated via electrophoresis. For the subsequent analysis of 306 antibodies, 1 × 20 µg of total protein was required, while only 1 × 10 µg of total protein was used for the measurement of 102 antibodies in the independent validation cohort. Size-separated proteins were blotted to a PVDF membrane and biotinylated. Every lane of the membrane was cut into 96 strips of 0.5 mm width, each corresponding to a certain molecular weight fraction. Each biotinylated protein strip was then placed in a specific well of a 96-well plate and elution buffer was added. The eluted proteins were incubated with magnetic color-coded beads (Luminex, Austin, USA) coated with neutravidin. The biotinylated proteins bind to the neutravidin beads such that each bead color represents proteins of one specific molecular weight fraction. The beads were mixed in pools of 96 bead identifies, thus resulting in a reconstitution of the original lane. For each protein measured, a small aliquot of the bead pool was incubated with a specific antibody and phycoerythrin-labeled secondary antibodies were added to generate signals.

Samples were read on a FlexMAP 3D flow cytometer (Luminex, Austin, USA), resulting in 96 values per antibody and sample, represented as graphs. Signal intensity was plotted against molecular weight and protein bands were visualized as peaks. While the molecular weight of each antibody was provided, an algorithm was used to identify adjacent peaks. The detected signals corresponded to the integral of the area of a peak, after subtraction of the local background. In a dedicated set-up, extraction of 4–16 FFPE samples plus DigiWest for up to 300 antibodies plus data analysis can be conducted within 10–12 days.

### Antibody selection

Antibody selection was performed on the set of 25 paired fresh frozen and FFPE tumor samples. From our collection of >1200 antibodies that had been pre-validated for DigiWest in fresh frozen materials, a selection of 634 antibodies was initially measured by DigiWest in 16 FFPE samples (3 HNSC, 4 LUSC, 3 LUAD, 3 COAD, and 3 PAAD). These 634 antibodies covered a broad range of molecular weights, targeting proteins and phosphoproteins in the cytoplasm and the nucleus. Among them, 306 antibodies were detectable in at least four samples or all samples of the same tumor type. These 306 antibodies were subsequently measured in the corresponding 16 fresh frozen samples and the additional nine pairs of fresh frozen and FFPE samples (2 HNSC, 1 LUSC, 2 LUAD, 2 COAD, and 2 PAAD).

A noticeable cross-reaction of the anti-rabbit secondary antibody (dk-α-rb-IgG (H + L)-RPE #711-116-152 Jackson, Westgrove, USA) was observed at 47–53 kDa in all HNSC samples, resulting in a stronger signal in fresh frozen than in FFPE samples. Therefore, we excluded a priori three antibodies (Cytokeratin 16, PPAR alpha-pS12, DAPK3 (ZIPK)-pT265) for which it was not possible to distinguish the specific signal from a cross-reaction. This resulted in a dataset of 303 antibodies.

Pearson correlations were computed for these 303 antibodies between fresh frozen and FFPE samples. Multiple testing correction for the significance of correlation scores was performed with the Benjamini–Hochberg (BH) method. Of the 303 antibodies, 121 showed a significant correlation (*p*-BH < 0.05), corresponding to 128 of 407 detected signals. In the cases in which more than one signal was detected for a given antibody, only the signal with the highest correlation factor was retained for further analysis.

Among these 121 antibodies, 12 were excluded because the detected signal shifted over 20% of the expected molecular weight of the antibody, another five antibodies were discarded because the peaks were not clearly identifiable, and two antibodies were excluded due to limited availability or redundancy.

In total, 102 of 634 antibodies (Supplementary Table 1) resulted in clear peaks at the expected molecular weights (±20%) and showed a significant correlation between the DigiWest signals detected in fresh frozen and FFPE samples. These 102 antibodies were subsequently used for cancer classification. Furthermore, DigiWest analysis in the independent validation cohort of 25 FFPE samples was also conducted with these 102 antibodies.

### Statistical analysis, classification, and data visualization

The analysis of processed DigiWest data was performed using the statistical programming language R [40] including the packages *gplots*, *kernlab, e1071*, and *caret* [41–44]. The data were transformed into log2 scale, and Pearson correlation coefficient was applied for the analysis between log2 expression values in fresh frozen and FFPE samples. The significance of correlation was assessed with the R-function *cor.test*. Heatmaps were generated based on the average-linkage method and Pearson correlation coefficient as similarity measure.

Radial basis function kernel support vector machines (SVM) were used as tumor classifiers. The model for the paired set was tuned and evaluated using nested cross-validation [45] with fivefold outer and fourfold inner cross-validation, repeated ten and five times, respectively. This ensures that no information from the validation samples was used for model selection at any point. The tuning parameters were chosen between *C* = 10^{0,1,2,3}^ and *σ* = 0.01 × 10^{−3,−2,…,3}^. The classifier used on the independent validation set was trained on the 25 FFPE samples from the paired set using fivefold cross-validation (ten repeats) and the same tuning parameters. The optimal parameters were *σ* = 0.0001 and *C* = 100. The SVM classifiers were compared with a random forest classifier, which yielded inferior classification accuracy for these prediction tasks.

The significance of differential expression between two groups was assessed with the *t*-test (R-function *pairwise.t.test* with default parameters). Multiple testing correction was performed with the BH method [46]. *p* values < 0.05 were considered statistically significant.

## Results

After the establishment and optimization of a DigiWest-compatible extraction protocol for FFPE samples, we determined the performance of DigiWest in FFPE tissue for the initial 634 antibodies. From these, we selected 303 antibodies that were expressed in at least four samples or all samples of the same tumor type to compare their signals in fresh frozen and FFPE samples (see “Methods”). Those 303 antibodies were measured in the 25 pairs of fresh frozen and FFPE primary tumor samples, which included five samples for each of the following tumor types: HNSC, LUSC, LUAD, COAD, and PAAD.

**Fig. 1:**
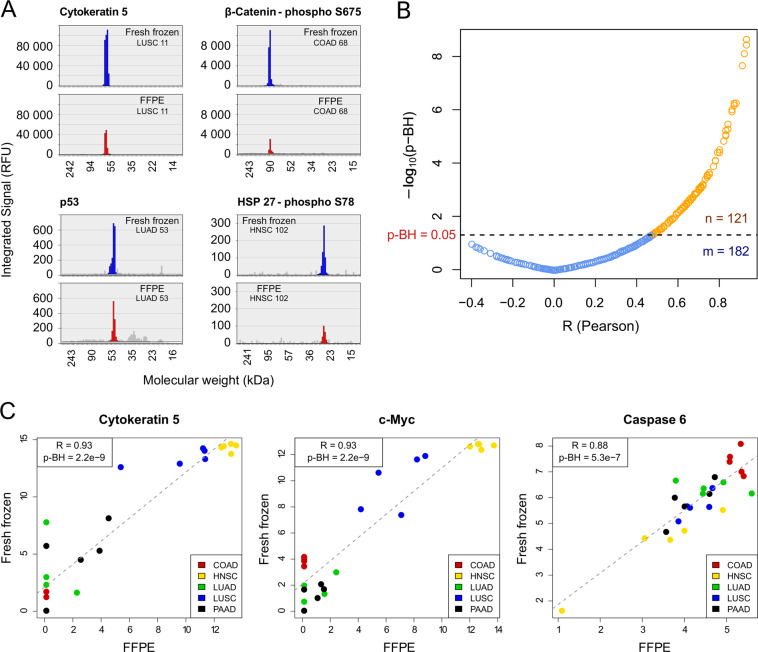
DigiWest protein profiling in fresh frozen and FFPE tissue. **a** DigiWest data displayed for four antibodies (Cytokeratin 5, p53, β-Catenin-phospho S675, HSP 27-phospho S78) in different tumor samples (fresh frozen in blue and FFPE tissue in red) with signal intensity (RFU: relative fluorescence units) plotted against molecular weight. **b** Pearson correlation coefficients (*R*) were computed for each antibody between all signals detected in both fresh frozen and FFPE samples. *p* values were corrected for multiple testing with the Benjamini–Hochberg method (*p*-BH), using a significance level of 0.05. The corresponding volcano plot shows the *p* values (−log10 transformed) plotted against correlation coefficients (*R*) (*n*: number of antibodies with significant correlation (*p*-BH < 0.05), colored in orange; *m*: number of antibodies with *p*-BH > 0.05, colored in blue). **c** Relative signals (log2) detected by DigiWest for three antibodies (Cytokeratin 5, c-Myc, Caspase 6) in 25 tumor samples, in both fresh frozen and FFPE samples. COAD colorectal adenocarcinomas, HNSC head and neck squamous cell carcinomas, LUAD lung adenocarcinomas, LUSC lung squamous cell carcinomas, PAAD pancreatic adenocarcinomas.

Then we investigated the correlations of all detected signals between fresh frozen and FFPE samples via Pearson correlation coefficients (*R*) to identify those antibodies that performed similarly in both tissue types. Figure 1 presents selected proteins comparing fresh frozen and FFPE signal intensities (Fig. 1a). The detected signals correspond to the integrated area of a peak at a certain molecular weight. If more than one signal was detected for a given antibody, only the signal with the highest correlation coefficient was included. Without applying a multiple testing correction, 150 out of the 303 antibodies (50%) were found to be significantly correlated between fresh frozen and FFPE tissue. After multiple testing correction (BH method), 121 antibodies (40%) demonstrated a significant correlation (*p*-BH < 0.05), with a correlation coefficient of *R* ≥ 0.47 (Fig. 1b). Nineteen of these antibodies were excluded because the peak was shifted (>20% of the expected molecular weight), or peaks were not clearly identifiable. Cytokeratin 5 and c-Myc showed the highest correlation coefficient among all proteins, with *R* = 0.93 and *p*-BH = 2.2e − 9. The detected signals for these proteins were particularly high in HNSC and LUSC, for both fresh frozen and FFPE tissue (Fig. 1c). The remaining 182 antibodies (60%) were not significantly correlated (*p*-BH > 0.05) and thus excluded. In total, 102 antibodies (34%) were used for further analysis. These 102 analytes comprised 14 out of 48 antibodies against phosphoproteins (29%) and 88 out of 255 against total proteins (35%). In fresh frozen samples, the mean signal intensities (log2 scale) were generally higher, except for one of 102 antibodies, and the signals were detected more frequently than in FFPE tissue (23 vs. 18 tumor samples on average).

**Fig. 2:**
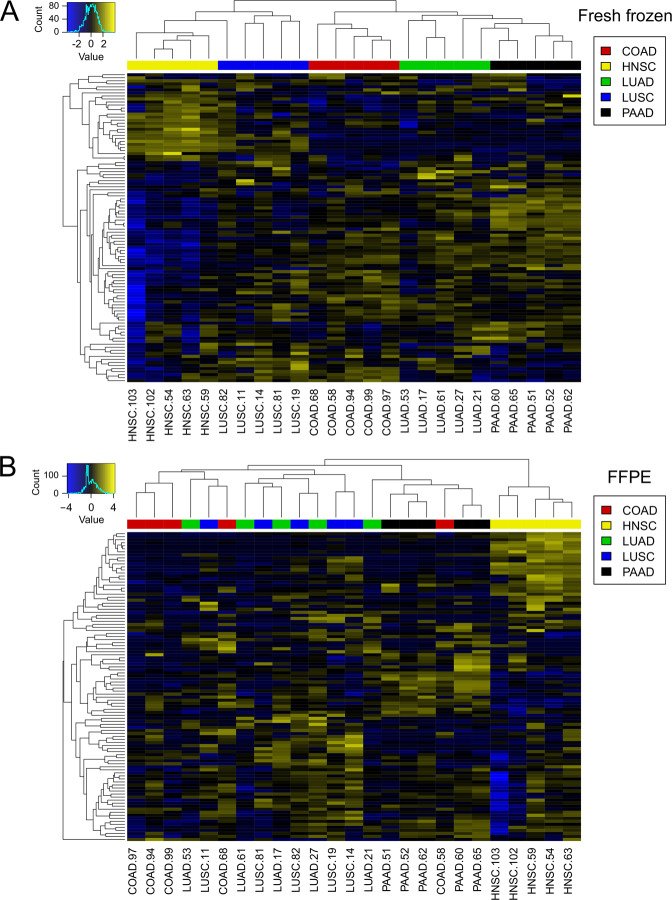
Heatmap and hierarchical clustering of DigiWest data. Overall, 102 antibodies were analyzed in 25 tumor samples, including five tumor types (**a** fresh frozen tissue; **b** FFPE specimens) with columns = tumor samples and rows = antibody signals. The color gradient from blue to yellow corresponds to low or high antibody-specific signals among the 25 tumor samples. COAD colorectal adenocarcinomas, HNSC head and neck squamous cell carcinomas, LUAD lung adenocarcinomas, LUSC lung squamous cell carcinomas, PAAD pancreatic adenocarcinomas.

Furthermore, we explored to which extent the selected panel of 102 antibodies would qualify to classify cancers with respect to their histological type and organ origin in the paired set. We first applied an unsupervised learning approach, visualizing DigiWest data by heatmaps combined with hierarchical clustering in both fresh frozen and FFPE tissue (Fig. 2). The 25 tumor samples were grouped together based on the correlation between the antibody signals. For fresh frozen samples, the different tumor types formed relatively distinct clusters. Only one LUSC was more closely grouped with HNSC than with the other LUSC. In the same way, two LUAD were more similar to PAAD than to the other LUAD. The two main clusters clearly separated adenocarcinomas from squamous cell carcinomas. In FFPE specimens, HNSC samples formed a distinct group, with high signals for a considerable number of proteins. All other tumor types were included in a second cluster, in which the tumor types were not well separated. Particularly, most of the LUAD and LUSC clustered together. Overall, the hierarchical clustering revealed clear differences between the investigated tumor types, especially in fresh frozen samples.

**Fig. 3:**
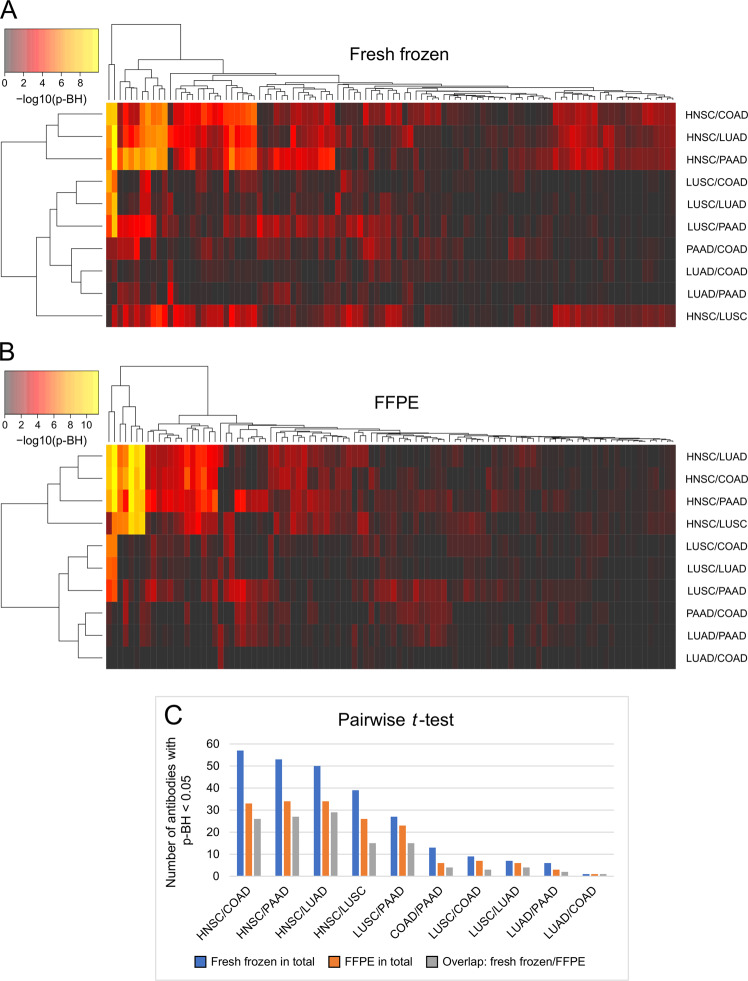
Pairwise *t*-test performed for 102 antibodies for each pair of tumor types. Heatmaps show *p* values (−log10 transformed) of the pairwise *t*-test (**a** fresh frozen and **b** FFPE tissue) after Benjamini–Hochberg (BH) correction. Columns represent the tested antibodies, and rows indicate pairs of tumor types. **c** Bar chart of the number of antibodies with a significant *p* value (*p*-BH < 0.05) in the pairwise *t*-test for each pair of tumors; comparison of fresh frozen samples, FFPE tissue, and overlap of both tissue types. COAD colorectal adenocarcinomas, HNSC head and neck squamous cell carcinomas, LUAD lung adenocarcinomas, LUSC lung squamous cell carcinomas, PAAD pancreatic adenocarcinomas.

A *t*-test was carried out in fresh frozen and FFPE samples for each pair of tumor types to identify the proteins that were suitable for discrimination of the respective tumor types. The corresponding *p* values were visualized in a heatmap, displayed in Fig. 3. Generally, more proteins with a significant *p* value (*p*-BH < 0.05) were found in fresh frozen than in FFPE tissue (Fig. 3a, b). Furthermore, a large proportion of the proteins that were significantly differentially expressed between the different tumor types in FFPE tissue were also significantly differentially expressed in fresh frozen samples (Fig. 3c). The expression of two proteins, Cytokeratin 5 and c-Myc, was significantly different between all squamous cell and adenocarcinomas, both in fresh frozen and in FFPE samples. Among all pairs of tumor types, those with the largest number of differentially expressed proteins all involved HNSC samples. For the three pairs of HNSC and adenocarcinomas (COAD, PAAD, and LUAD), at least 50 proteins in fresh frozen tissue and 33 in FFPE samples were significantly different. Both types of squamous cell carcinomas (HNSC and LUSC) could also be discriminated by a considerable number of proteins (39 in fresh frozen and 26 in FFPE tissue). In contrast, only a few proteins were suitable for distinguishing LUSC vs. LUAD, as well as between two types of adenocarcinomas. For LUAD vs. COAD, only one protein with a significant *p* value was determined (Thyroid transcription factor-1 (TTF-1)).

**Fig. 4:**
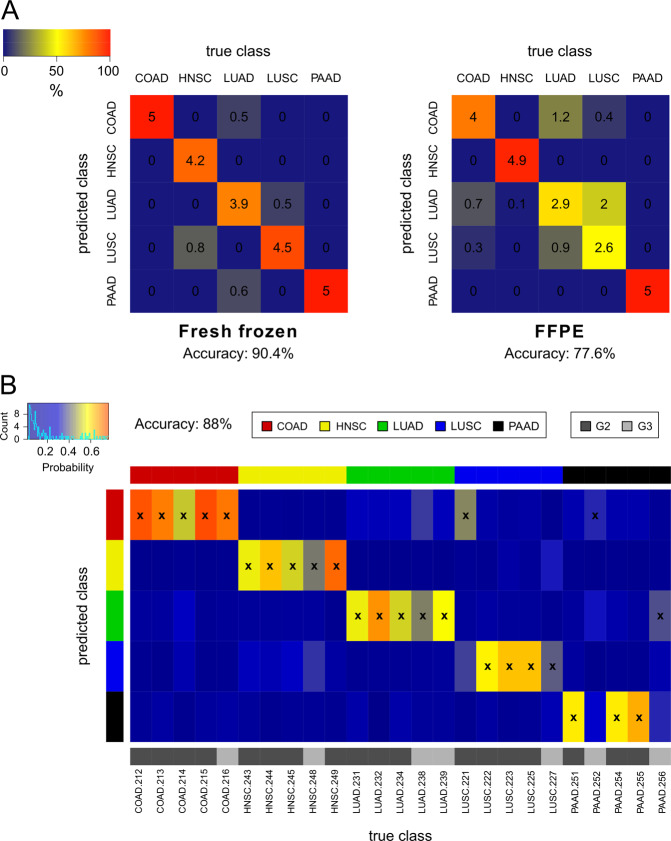
Multiclass cancer classification by machine learning. **a** Contingency matrices showing the classification accuracy of the SVM-based models obtained by repeated nested cross-validation with 25 tumor samples in fresh frozen and FFPE tissue. The numbers indicate how many of the five samples of each tumor type are classified on average in each class. The corresponding percentages are visualized by the color scheme (blue: low, red: high). **b** Classification results of the SVM algorithm in an independent validation cohort of 25 FFPE samples with five cases per tumor type. The predicted tumor type is marked with a cross; the color gradient indicates the confidence of the SVM classifier for each class (blue: low probability, red: high probability). Histological grades: G2 moderately and G3 poorly differentiated tumor samples. COAD colorectal adenocarcinomas, HNSC head and neck squamous cell carcinomas, LUAD lung adenocarcinomas, LUSC lung squamous cell carcinomas, PAAD pancreatic adenocarcinomas.

To classify the tumor samples according to their tissue of origin, an SVM algorithm with repeated nested cross-validation (fourfold internal and fivefold external) was applied to the set of paired tumor samples. With the resulting classification, an overall accuracy of 90.4% was obtained in fresh frozen samples (standard deviation of 5.4% over ten repeats; Fig. 4a). All colorectal and PAAD were classified correctly. The lowest accuracy of 78% was attained for LUAD. For FFPE samples, the SVM classifier yielded an overall accuracy of 77.6% (standard deviation of 3.4% over ten repeats). The individual accuracies among the different tumor types varied more in FFPE than in fresh frozen specimens. On the one hand, all PAAD and almost all HNSC (98%) were correctly assigned to their tissue of origin. On the other hand, the classifier based on FFPE data was often not able to discriminate between LUSC and LUAD. Only 52% of LUSC were predicted correctly, while 40% of LUSC were misclassified as LUAD. Similar results were obtained for LUAD, with 58% of the samples being correctly classified and 18% of LUAD categorized as LUSC. If squamous cell and adenocarcinomas of the lung were considered as only one tumor type since they arise in the same organ, the accuracy of this class increased to 84%. This resulted in an overall accuracy of 89.2%. Furthermore, some misclassification of FFPE tumor samples also occurred between LUAD and COAD.

In general, the classification model based on fresh frozen samples performed better than the classifier constructed with FFPE samples (overall accuracies of 90.4% vs. 77.6%). Both models performed best for PAAD and yielded there a prediction accuracy of 100%. For LUSC, LUAD, and COAD, the accuracy was at least 20% higher in fresh frozen than in FFPE samples. In contrast, the classification of HNSC yielded better results with FFPE samples.

Finally, we tested the performance of the SVM classifier in an independent validation cohort of 25 FFPE primary tumor samples, containing five cases per tumor type. This yielded an overall accuracy of 88%. The classification results and their probability scores are visualized in Fig. 4b. All COAD, HNSC, and LUAD cases were correctly assigned to their tissue of origin. In total, only three tumor samples were misclassified. One LUSC and one PAAD were categorized as COAD, while another PAAD was classified as LUAD. Concerning the histological grade, 17 out of 18 moderately (G2) and five out of seven poorly (G3) differentiated tumor samples were correctly predicted. The mean probability scores for the correct class in poorly differentiated cases were slightly lower than in moderately differentiated tumor samples (mean 0.45 vs. 0.61, *p* = 0.03). Overall, the SVM classifier performed better in the independent validation cohort than in the initial 25 FFPE samples from the paired set (88% vs. 77.6% overall accuracy).

## Discussion

Protein analysis in FFPE tissue is known to be challenging, as formaldehyde fixation induces cross-links, and proteins must be recovered before the analysis. In this study, we demonstrated that DigiWest multiplex protein analysis is feasible in FFPE samples and can be used for diagnostic cancer classification. By comparing the performance of 303 antibodies in fresh frozen and FFPE tissue, we identified 102 antibodies that yielded sound and comparable results in both. Importantly, this also included 14 antibodies against phosphoproteins, which allows getting better insights into protein activation and oncogenic signaling.

We identified proteins that were best suited for the pairwise distinction of tumor types. Some of these proteins are well known to be relevant in the corresponding tumor types. Exemplarily, we found that TTF-1 was highly expressed in many LUAD and was a useful protein to differentiate LUAD from every other tumor type both in fresh frozen and in FFPE tissue, consistent with its wide use in diagnostics. TTF-1 is usually used in immunohistochemistry to identify LUAD and to discern primary tumors of the lung from metastases [47]. According to our results, both Cytokeratin 5 and c-Myc were able to distinguish squamous cell carcinomas from adenocarcinomas, and their signal was particularly strong in LUSC and HNSC. Cytokeratin 5 is commonly used as an immunohistochemical marker for squamous cell carcinomas [48]. However, although c-Myc is often expressed in those tumor types, it is not known to be a specific marker for squamous cell carcinomas and has been detected in other tumor types before [49–52]. The expression of these proteins might not only be useful for diagnostic purposes but could further insight into the tumor’s biology and indicate potential therapeutic targets.

Based on the 102 selected antibodies, we developed two approaches for cancer classification in the set of paired tumor samples. First, an unsupervised hierarchical clustering method and second a supervised SVM algorithm, which classified the tumors according to their tissue of origin. Both methods achieved a better distinction between the five tumor types in fresh frozen samples than in FFPE tissue. This is probably due to a higher sensitivity of DigiWest analysis in fresh frozen samples. Although the same 102 antibodies were used in both tissue types, the signals were generally detected more frequently in fresh frozen than in FFPE samples. Furthermore, the mean signal intensities were higher, and more proteins were suited for the pairwise distinction of tumor types in fresh frozen samples. Overall, the SVM algorithm reached an accuracy of 90.4% in fresh frozen and 77.6% in FFPE samples.

The reduced overall accuracy in FFPE samples from the paired set was mainly due to misclassification between squamous cell and adenocarcinomas of the lung, which accounted for 11.6% of all errors. These tumor types are closely related, arise in the same organ, and nonneoplastic cells may contribute to the signal. This could make them more difficult to distinguish, even though it does not fully explain the lower accuracy for lung cancer in FFPE compared with fresh frozen samples. If LUSC and LUAD were considered as only one cancer type, the overall accuracy of the FFPE classifier increased to 89.2%, which is very close to that in fresh frozen specimens.

Of note, in the independent validation cohort of 25 FFPE samples, the SVM classifier reached an overall accuracy of 88%, which is slightly higher than in the FFPE samples from the paired set (77.6% overall accuracy). This is likely to be due to the larger number of training samples available to the classifier (25 instead of the 20 samples used in the nested cross-validation of the paired set). Furthermore, it was associated with a better distinction between LUSC and LUAD. The SVM classifier was also applicable to poorly differentiated tumor samples. Five of the seven poorly differentiated tumor samples were correctly assigned to their tissue of origin. However, as expected from tumor biology, the probability scores for the correct class of poorly differentiated tumor samples were, on average, slightly lower than those of moderately differentiated cases.

Our classifier was able to distinguish with high accuracy between squamous cell carcinomas of different origins. In a previous study, Bohnenberger et al. [17] developed a classifier based on quantitative mass spectrometry data in FFPE samples, which differentiated between HNSC and LUSC with an accuracy of 86.8% in an independent test set [17]. However, more than 1100 proteins were necessary to achieve these results, and the accuracy decreased to 76.8% when only 100 proteins were included [17]. We demonstrated, based on the data of 102 antibodies, that it is possible to generate a classifier with comparable accuracy for discriminating five tumor types (77.6% accuracy in the paired set and 88% in the independent validation cohort) with DigiWest multiplex protein profiling. In our study, no misclassifications occurred between LUSC and HNSC in FFPE samples. As patients with a primary HNSC often develop distant metastasis in the lungs, but at the same time have an increased risk for the occurrence of a second primary tumor of the lungs, the classifier might be used to complement current diagnostic methods.

The overall accuracy of our classifier is comparable with those of previous studies establishing a multiclass cancer classifier based on protein profiles, even though those had only been carried out on fresh frozen samples. On the one hand, our classifier performed slightly better in fresh frozen samples (90.4% accuracy) than two studies that each classified six types of adenocarcinomas using MALDI mass spectrometry with an average accuracy of 82% [20, 21]. On the other hand, the accuracy of our classifier was slightly lower than that of Zhang et al., which reached an accuracy of 93.6% [22]. However, they used a considerably larger number of samples for training, originating from RPPA data of ten tumor types from The Cancer Proteome Atlas [22]. While this accuracy is relatively high, RPPA is not applicable in the typical clinical setting, as outlined below.

Altogether, our classifier achieved a high accuracy using only a modest number of samples for training. A greater number of samples might better represent various differentiation statuses or molecular subtypes within each tumor type, possibly leading to increased accuracy.

A further advantage of DigiWest analysis is that only a low amount of material is required, as 20 µg of protein was sufficient for the measurement of 303 antibodies (resp. 10 µg for 102 antibodies). This makes DigiWest particularly useful for clinical samples, which are often limited in their amounts and routinely collected as FFPE tissue. RPPA represents another approach for the multiplex analysis of proteins, with the advantage that several hundreds of samples can be measured in parallel [25]. However, RPPA is less appropriate for the analysis of single or only few samples as is the case in routine diagnostics where samples have to be processed as they accrue for timely diagnoses. In contrast to RPPA, the proteins in DigiWest analysis are separated according to their molecular weight, which enables direct quality control of the raw data for each antibody in each sample to rule out unspecific signals. This step of identification and interpretation of the specific signals remains critical, as seen in the above-mentioned case of anti-rabbit secondary antibody cross-reaction at 50 kDa in HNSC samples. This emphasizes the need for a careful selection of antibodies.

In summary, our study demonstrates that DigiWest multiplex protein profiling can be performed on FFPE tissue specimens. We identified 102 antibodies against a variety of proteins and phosphoproteins that showed expression in both fresh frozen and FFPE samples, as well as correlating signals between the two. These 102 analytes were used to develop an SVM algorithm capable of classifying samples of five tumor types according to their tissue of origin with high accuracy in both fresh frozen and FFPE samples. Thus, DigiWest analysis constitutes a promising approach for analyzing the protein expression of FFPE samples, which can be used for diagnostic cancer classification and might also help to identify novel biomarkers or potential therapeutic targets in the future.

## Supplementary information

Supplementary Table 1
